# Norcantharidin inhibits TOP2A expression via H3K27me3 mediated epigenetic regulation to alleviate the progression of hepatocellular carcinoma

**DOI:** 10.3389/fphar.2025.1541298

**Published:** 2025-04-03

**Authors:** Ruibing Wu, Hengye Yuan, Yuehua Wang, Xianggang Gou, Wanhua Hou, Zhongzheng Zhou, Xinran Wang, Xiuling Deng, Changshan Wang, Haisheng Wang, Jia Yan

**Affiliations:** ^1^ School of Basic Medicine, Inner Mongolia Medical University, Hohhot, Inner Mongolia, China; ^2^ College of Life Science, Inner Mongolia University, Hohhot, Inner Mongolia, China; ^3^ Medical Experimental Center of Basic Medical School, Inner Mongolia Medical University, Hohhot, Inner Mongolia, China

**Keywords:** NCTD, TOP2A, H3K27me3, EZH2, HCC

## Abstract

**Background:**

Norcantharidin (NCTD), a bioactive compound derived from traditional Chinese medicine, has demonstrated promising anticancer activity against multiple malignancies, particularly hepatocellular carcinoma (HCC). However, its epigenetic regulatory mechanisms and associated transcriptional consequences remain poorly characterized.

**Methods:**

In this study, we integrated biochemical assays with a panel of cellular analyses assessing cell viability, proliferation, colony formation, and migratory capacity to investigate NCTD’s therapeutic potential in HCC progression. Potential molecular targets of NCTD were systematically identified through integrated network pharmacology approaches. Chromatin immunoprecipitation quantitative PCR (ChIP-qPCR) was performed to quantify H3K27me3 enrichment level at the TOP2A locus in NCTD-treated HCC cells. Molecular docking simulations were employed to examine structural interactions between NCTD and EZH2 (enhancer of zeste homolog 2), while co-immunoprecipitation assays were further conducted to validate protein-protein interactions between EZH2 and protein phosphatase 1 (PP1).

**Results:**

We identified topoisomerase IIα (TOP2A) as a critical molecular target mediating NCTD’s anti-HCC effects. Functional characterization revealed that NCTD significantly attenuated HCC cell proliferation and induced G2/M phase cell cycle arrest through disruption of the TOP2A-p53 signaling axis. Mechanistic investigations demonstrated that NCTD epigenetically suppresses TOP2A transcription via PRC2 (Polycomb Repressive Complex 2)-mediated deposition of the repressive histone mark H3K27me3 at the TOP2A promoter. Structural biology analyses confirmed direct binding of NCTD to EZH2 protein, consequently impairing PP1-mediated dephosphorylation and enhancing PRC2 complex stability.

**Conclusion:**

Our findings establish that NCTD exerts anticancer effects in HCC through epigenetic silencing of TOP2A. This work not only elucidates a novel pharmacoepigenetic mechanism underlying NCTD’s antitumor activity but also provides translational rationale for developing PRC2-targeted therapeutic strategies in HCC management.

## Introduction

Cantharides, a traditional Chinese medicine with a lengthy history in cancer therapy, owes its efficacy primarily to its active compound, cantharidin (CTD). Extensive research has documented the anticancer properties and immune-enhancing capabilities of CTD associated pharmaceutical preparations, particularly in the treatment of various solid tumors, especially liver cancer (Li et al., 2023; Naz et al., 2020; Ren and Kinghorn, 2021). Notably, Chinese medicine preparations enriched with cantharidin, such as Aidi injection, have demonstrated significant antitumor effects and have been clinically approved in China since 2002 (Yang et al., 2022). Multiple studies have illuminated the underlying mechanisms behind these effects, revealing their intricate involvement in multi-channel and multi-target interactions. These interactions encompass the inhibition of cell proliferation, autophagy, migration, and metastasis, alongside the induction of apoptosis and the enhancement of anticancer immunity (Yan et al., 2023a; Song et al., 2020; Wang CH. et al., 2022). Furthermore, CTD has been reported to regulate the crucial signaling pathways such as *JAK2*/*STAT3*/*TWIST*, *PI3K*/*Akt*, and *Wnt/β-catenin*, as well as *MZF1/c-MYC* axis, thereby impairing cancer cell development (Wang CH. et al., 2022; Zhu et al., 2020; Pang et al., 2022).

In the pursuit of mitigating the toxicity associated with CTD, a demethylated analogue known as norcantharidin (NCTD) was developed for application in cancer therapy. NCTD, which retains the antitumor efficacy of CTD while significantly reducing its adverse effects, represents a significant advancement in the field (Qian et al., 2023; Zhai et al., 2022). This compound exhibits its antitumor properties by targeting the same pathways previously implicated with CTD, thereby effectively suppressing the progression of HCC. Furthermore, NCTD has demonstrated the ability to enhance the antitumor activity of sorafenib, a commonly used therapeutic agent in HCC, by inhibiting the IL-6/STAT3 signaling pathway (Yousef et al., 2023). Despite these promising findings, there remains a critical need for more comprehensive and systematic studies to elucidate the molecular mechanisms underlying the antitumor effects of NCTD. Such research is imperative for advancing our understanding of NCTD’s therapeutic potential and facilitating its translation into clinical practice.

NCTD has been established as a protein phosphatase inhibitor, presenting itself as a potential target for its anticancer activities (Li et al., 2010). Our previous research found that the expression of TOP2A was suppressed by CTD in HCC cells (Yan et al., 2023b). Indeed, TOP2A, highly expressed in various malignancies, has a critical role in the initiation and progression of cancer. It has been demonstrated that TOP2A is associated with tumour proliferation, metastasis and resistance to chemotherapy (Zhang et al., 2020; Wang Z. et al., 2022). It has been implicated in enhancing HCC metastasis through the mediation of the p-ERK1/2/p-SMAD2/Snail pathway, and its inhibition has been shown to reverse drug resistance to regorafenib in HCC (Zhang et al., 2020). Furthermore, cDCBLD2 could bind to miR-345–5p, thereby enhancing the stability of TOP2A mRNA as a miRNA sponge. This interaction subsequently diminishes the cytotoxic effects of sorafenib on HCC (Ruan et al., 2023). Additionally, previous studies have identified a dual upregulation of EZH2 and TOP2A during cancer progression. Furthermore, it has been reported that the EZH2-H3K27me3-mediated silencing of mir-139–5p can activate TOP2A, thereby inhibiting cellular senescence in HCC (Wang et al., 2023). However, the regulatory mechanisms underlying the interplay between EZH2 and TOP2A in HCC treatment remain unclear.

Epigenetics alterations play a nonnegligible role in influencing gene regulation during the initiation and progression of tumors. Increasing studies suggest that TCM exhibits considerable antitumor effects through the modulation of epigenetic modifications (Xiang et al., 2019; Zhu et al., 2022). Notably, curcumin has been demonstrated to downregulate the expression of the PRC2 subunit EZH2 in various cancer cells (Ma et al., 2019). Additionally, lonicerin has been reported that it could target EZH2 to ameliorate ulcerative colitis through autophagy-mediated inactivation of the NLRP3 inflammasome (Lv et al., 2021). Scutellarein has been confirmed that it inhibits tumor growth and metastasis in ovarian cancer by regulating the EZH2/FOXO1 signaling pathway (Lang et al., 2021). Recently, our study indicates that cantharidin could suppresses HCC development by regulating EZH2/H3K27me3-dependent cell cycle progression (Yan et al., 2023b). However, several underlying mechanisms remain to be elucidated. In order to be more conducive to the later conversion application, we explored the molecular mechanism of NCTD targeting regulation of TOP2A expression through PRC2-associated epigenetic in HCC therapy. These findings underscore the potential of TCM in targeting epigenetic modifications for cancer therapy, warranting further investigation.

We conducted further research to investigate the molecular mechanisms underlying the effects of NCTD on HCC progression. Our hypothesis was that NCTD might target TOP2A to hinder the development and progression of HCC. In this study, we found that NCTD inhibits the biological functions of HCC cells by suppressing TOP2A expression. Mechanistically, NCTD suppresses TOP2A through affecting H3K27me3 mediated transcriptional regulation to induce cell cycle arrest by regulating p53 signaling axis, thereby inhibiting HCC progression. These results contribute to a better understanding of the molecular mechanisms of NCTD in HCC and may guide future therapeutic strategies.

## Results

### Norcantharidin inhibits the biological functions of HCC cell

**FIGURE 1:**
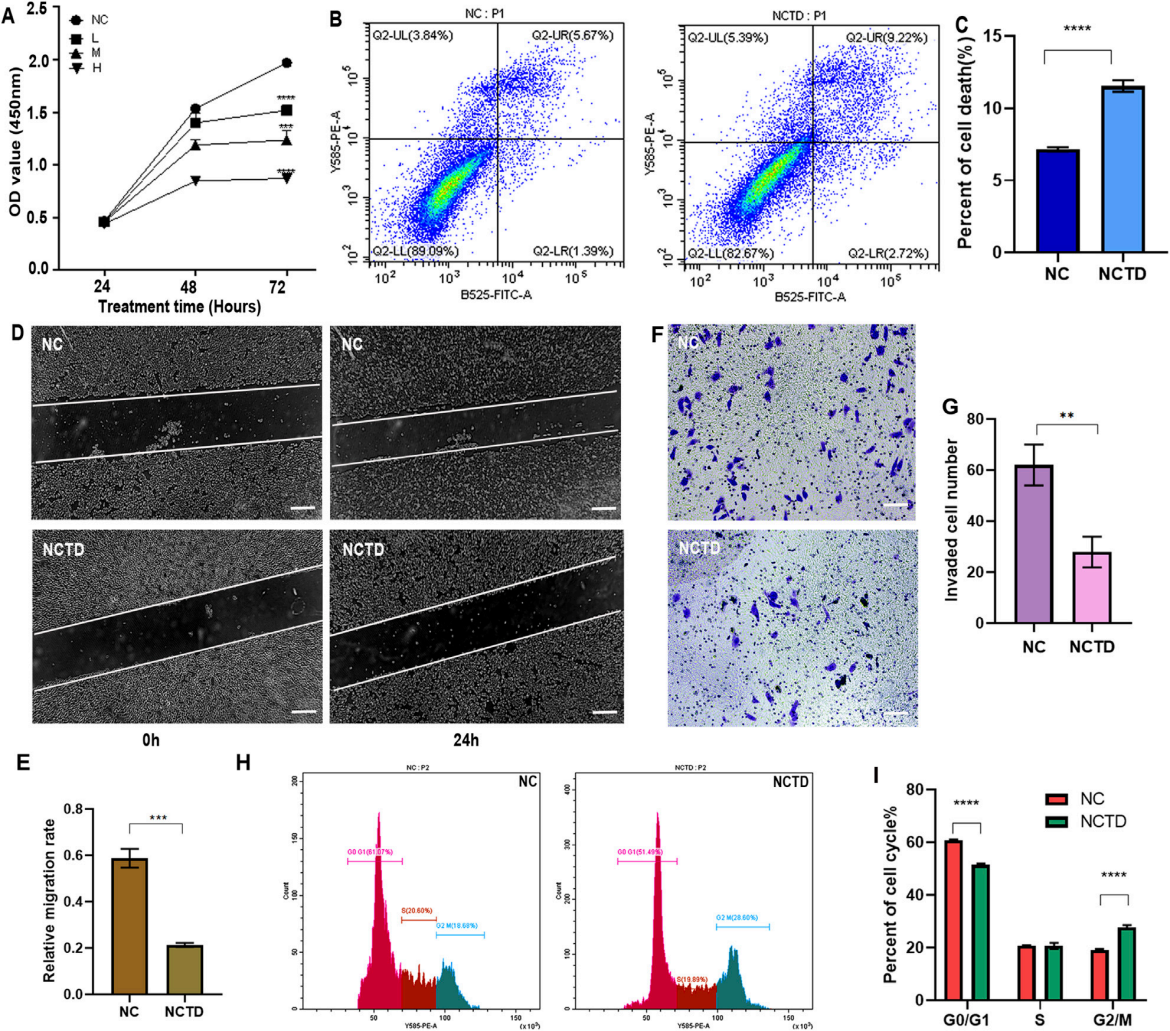
NCTD suppresses proliferation, migration, and invasion of HCC Cells. **(A)** NCTD inhibits HCC cell proliferation. The proliferation of HCC cell line HepG2 treated with NCTD was assessed using the CCK-8 assay. NC stands for negative control, which was treated with 0.1% DMSO, L group: low concentrations of NCTD (20 µM), M group: medium concentration (40 µM), and H group: high concentration (60 µM) **(B, C)** NCTD induces HCC cell apoptosis. **(B)** Flow cytometry was employed to quantify the apoptotic rate in HepG2 cells following NCTD treatment. Specifically **(C)** depicts the apoptotic rate measured via flow cytometry. **(D, E)** Cell migration capacity is suppressed by NCTD in HCC Cells. **(D)** The wound healing assay was performed to evaluate the migratory ability of HepG2 cells after NCTD exposure. Bars represent 100 µm. **(E)** Statistical plots compare cell migration efficiency before and after NCTD treatment in HepG2 cells. **(F, G)** Cell invasion ability is suppressed by NCTD in HCC Cells. **(F)** Transwell invasion assay was performed to evaluate the migratory ability of HepG2 cells treated with NCTD. Bars represent 100 µm. **(G)** Statistical plots compare cell invasion efficiency before and after NCTD treatment in HepG2 cells. **(H, I)** Cell Cycle analysis in NCTD-treated HCC Cells. Bars represent 100 µm. **(H)** The cell cycle was analyzed by flow cytometry in NCTD treated HepG2 cells. **(I)** Cell cycle statistical analysis results. All the Data were shown as mean ± SD. Statistical significance was indicated as follows, **p < 0.01, ***p < 0.001, and ****p < 0.0001. All experiments were repeated three times.

To validate the role of NCTD in HCC treatment, we evaluated its impact on the proliferative capacity of HCC cells using the CCK8 assay. The results demonstrated a significant reduction in cell proliferation following NCTD treatment, with a dose-dependent effect observed at low (20 µM), medium (40 µM), and high (60 µM) concentrations in HepG2 cell (Figure 1A). Furthermore, the cytotoxicity of NCTD in normal liver cells and other liver cancer cells, including Huh7 and MHCC97-H was also detected by the CCK8 assay. The results indicted that low concentrations of NCTD also showed cytotoxicity to other liver cancer cells, but no significant toxicity to normal liver cells (Supplementary Figure 1A-C). Additionally, flow cytometry analysis further revealed that NCTD promotes HepG2 cells apoptosis, as evidenced by an increased proportion of apoptotic cells (Figures 1B,C). The wound-healing and transwell assays showed a significant decrease in the migration and invasion abilities of NCTD-treated HepG2 cells (Figures 1D–G). Furthermore, NCTD treatment resulted in a significant arrest of the cell cycle in HCC cells (Figures 1H,I). Collectively, these findings demonstrate that NCTD effectively suppresses the proliferation, migration, invasion, and promotes apoptosis in HCC cells.

### TOP2A as a target of NCTD in biological function of HCC cells

**FIGURE 2:**
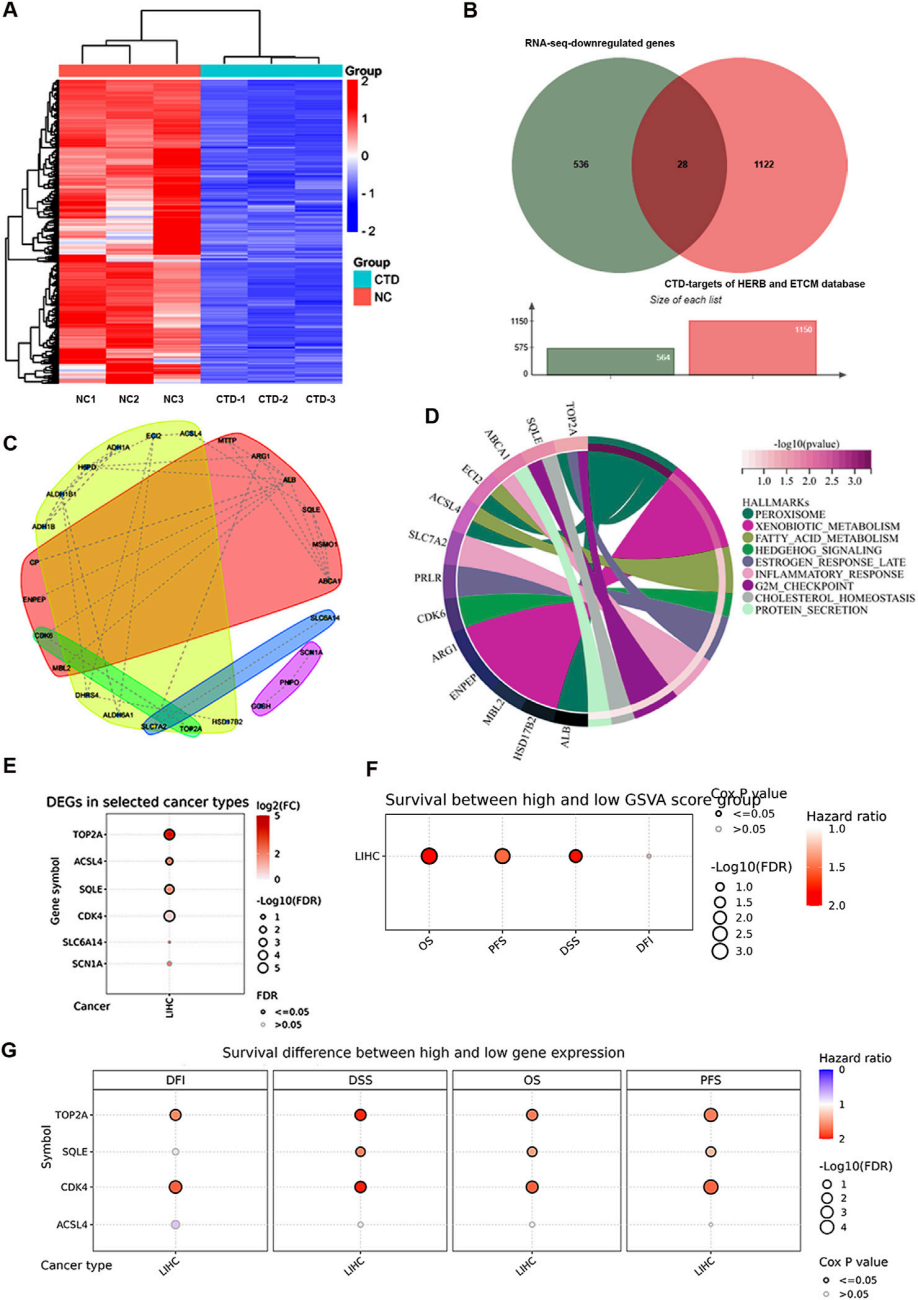
Multi-omics analysis of CTD-associated molecular targets in HCC. **(A)** Hierarchical clustering heatmap of CTD-regulated differentially expressed genes (log2FC < -2, FDR <0.05) were identified by RNA-seq analysis. **(B)** Venn diagram illustrating the intersection of CTD-associated targets from RNA-seq data and pharmacological databases (HERB and TCMSP). **(C)** Protein-protein interaction network of CTD target genes constructed using STRING database, with node colors representing distinct functional modules. The red and yellow clusters denote two major hub communities. **(D)** KEGG pathway enrichment analysis of CTD downregulated genes. **(E)** Validation of CTD-downregulated gene expression patterns in HCC clinical specimens using TCGA-LIHC dataset, the figure represents genes with elevated expression in LIHC. **(F)** Prognostic significance of CTD target gene set was assessed through survival analysis using GSCA platform (overall survival, disease-specific survival, recurrence-free survival, and disease-free interval). **(G)** Clinical correlation between the expression of representative CTD-regulated oncogenes and patient prognosis. *p*-value <0.05 was considered statistically significant.

Building upon our previous RNA-sequencing (RNA-seq) findings on CTD-mediated transcriptional regulation in HCC (Yan et al., 2023a), we performed an integrative analysis of CTD targets using multi-omics approaches. RNA-seq analysis result revealed that 565 genes were significantly downregulated (log2FoldChange < -2, adjusted *p*-value <0.05) upon CTD treatment, as visualized in the hierarchical clustering heatmap (Figure 2A). To identify clinically relevant targets of CTD, we cross-referenced these differentially expressed genes (DEGs) with CTD-associated targets from the HERB and TCMSP databases, identifying 28 consensus targets through Venn analysis (Figure 2B). Protein-protein interaction network construction using STRING database uncovered key hub genes involved in cell cycle regulation and metabolic reprogramming (Figure 2C). Consistent with these findings, Kyoto Encyclopedia of Genes and Genomes (KEGG) pathway enrichment analysis demonstrated that these genes were significant enrichment in cell cycle and metabolic pathways (Figure 2D). Notably, among the top-ranked CTD-suppressed genes, we found that five genes, including *TOP2A*, *ACSL4*, *SQLE*, *CDK4*, and *SLC6A14* were significantly elevated in HCC clinical specimens, suggesting that NCTD targets to inhibit the expression of these genes to exert anti-tumor effects in HCC (Figure 2E).

Clinical correlation analysis revealed that elevated expression of these genes was significantly associated with poor patient outcomes across multiple survival metrics: overall survival (OS, HR = 1.81, *p* = 7.49e-4), disease-specific survival (DSS, HR = 1.79, *p* = 9.77e-3), recurrence-free survival (RFS, HR = 1.79, *p* = 9.77e-3), and disease-free interval (DFI, HR = 1.24, *p* = 0.2) (Figure 2F). Furthermore, the expression of *TOP2A* and *CDK4* was dramatically associated with prognosis in HCC (Figure 2G). Collectively, these multi-dimensional analyses identify TOP2A as a target for HCC therapy through CTD-mediated transcriptional regulation.

**FIGURE 3:**
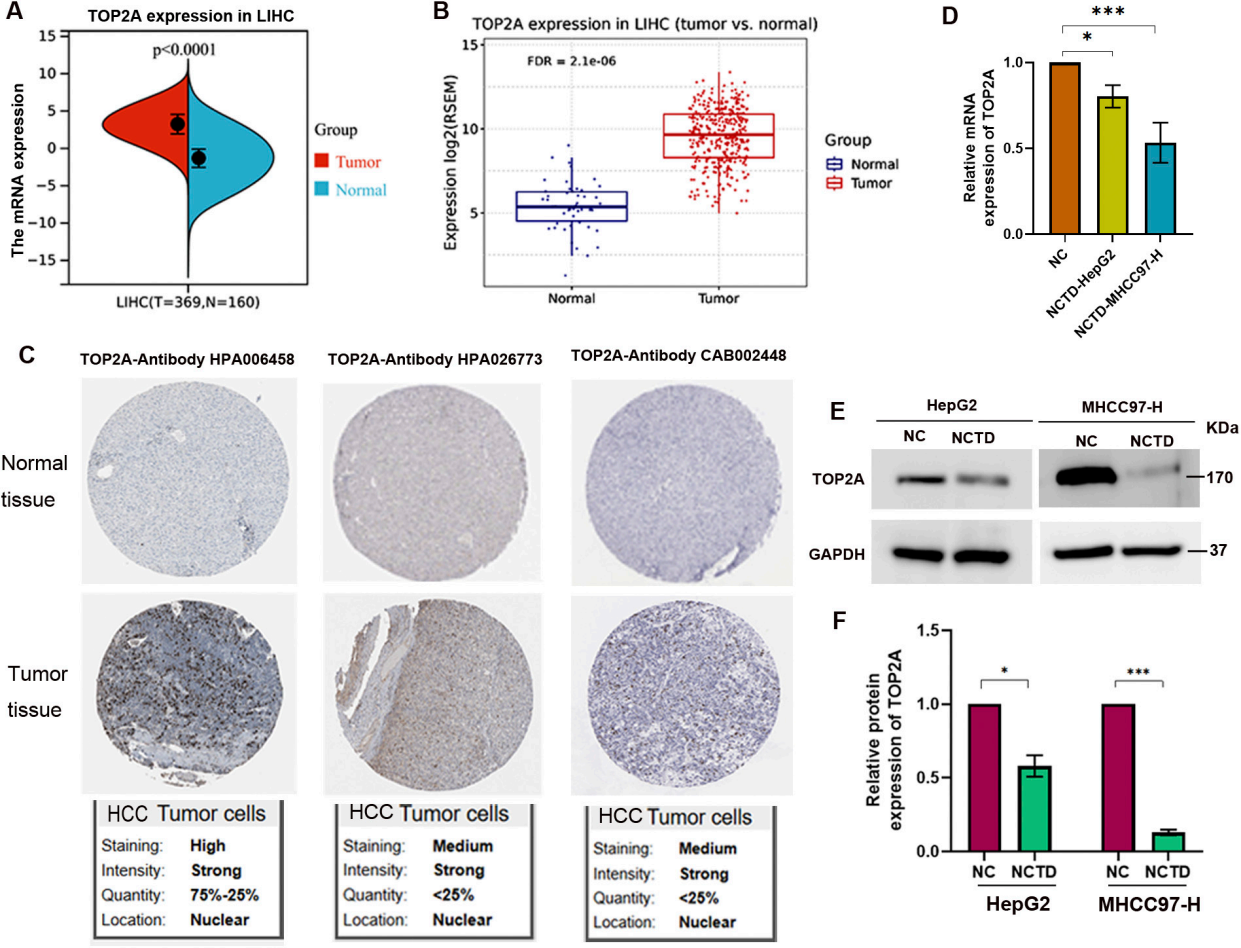
TOP2A is increased in HCC patients and its expression is downregulated by NCTD in HCC **(A, B)** Expression patterns of TOP2A in two cohorts of HCC patients. TOP2A expression was evaluated in HCC tissues. **(A)** Analysis of TOP2A expression in LIHC based on the TCGA database. Specifically, TOP2A expression was evaluated in HCC tissues (T = 369) compared to matched adjacent normal samples (N = 160), yielding a *p*-value <0.0001. **(B)** The validation results using the GSCA database for LIHC comprises 424 samples, including 374 tumor and 50 normal tissues (with 50 paired samples). RSEM-normalized mRNA expression data were sourced from the harmonized TCGA Atlas within UCSC Xena. Statistical significance was determined with an FDR (false discovery rate) ≤0.05. **(C)** IHC analysis substantiates the enhanced expression of TOP2A protein in HCC patient samples by The Human Protein Atlas database, utilizing three distinct antibody probes targeting TOP2A. The upper panel displays IHC staining of normal liver tissue, whereas the lower panel showcases IHC of tumor tissue, clearly indicating an increase in TOP2A protein levels in HCC. **(D)** NCTD treatment significantly downregulates TOP2A mRNA expression in HepG2 and MHCC97-H cells. **(E)** Western blot analysis showing NCTD-mediated suppression of TOP2A protein expression in HepG2 and MHCC97-H cells. **(F)** Quantitative analysis of protein band intensity of TOP2A normalized to GAPDH. All data are reported as mean ± SD. Significance levels are denoted as *p < 0.05, **p < 0.01, and ***p < 0.001. All experiments were conducted in triplicate to ensure reproducibility.

To further investigate the role of TOP2A in HCC, we compared its expression levels between HCC patients and normal individuals. The results revealed a significant increase in TOP2A mRNA expression in HCC patients (Figures 3A,B). In addition, we confirmed the expression of TOP2A in different cell lines compared with normal liver cell. The results indicated that the mRNA level of TOP2A was significantly increased in HCC cell lines (Supplementary Figure 2A). The protein expression of TOP2A also elevated in HCC cell lines (Supplementary Figure 2B, C). Furthermore, IHC analysis results indicated that TOP2A protein expression was elevated in HCC patients (Figure 3C). Therefore, TOP2A is involved in tumor progression as an oncogene, and NCTD likely target TOP2A to inhibit its expression, thereby inhibiting tumor progression.

To further elucidate the mechanisms underlying the inhibitory effect of NCTD on HCC, we examined the impact of NCTD on TOP2A expression. The mRNA and protein levels of TOP2A were assessed in NCTD-treated HCC cells, including HepG2 and MHCC97-H (Figures 3E,F). The results demonstrated a notable decrease in TOP2A expression, both at the mRNA and protein levels, following NCTD treatment in HCC cells. These findings indicate that TOP2A could be a crucial therapeutic target for NCTD in liver cancer, providing insights into the mechanisms by which NCTD exerts its antitumor effects in HCC.

### Overexpression of TOP2A rescued the inhibitory effect of NCTD on the biological activities of HCC cells

**FIGURE 4:**
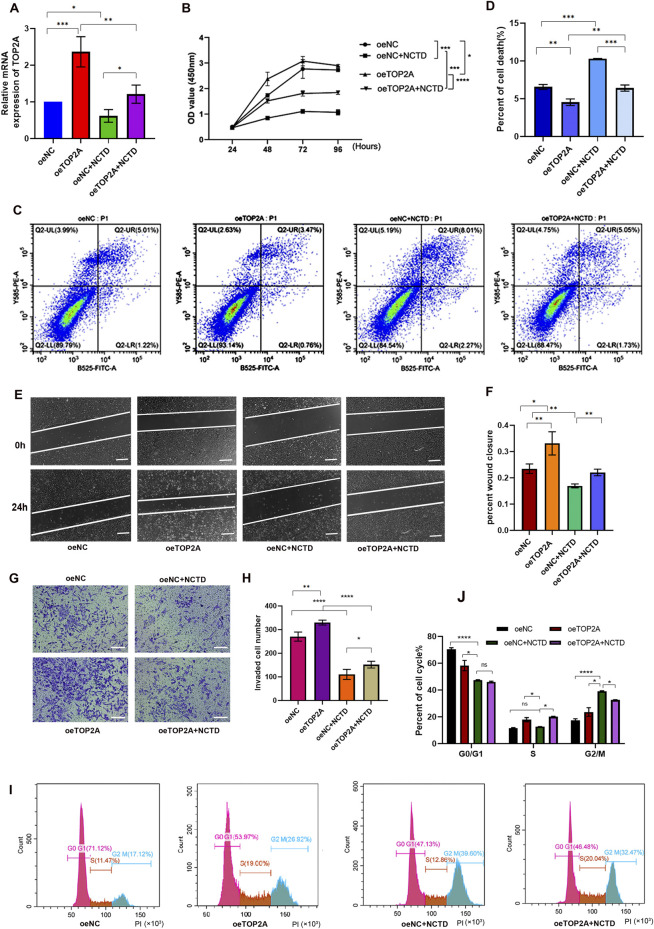
Overexpression of TOP2A reverses the inhibitory effects of NCTD on HCC cells. **(A)** The mRNA expression of TOP2A was confirmed using qPCR in different groups, including in HepG2 cell lines treated with NCTD, in cells that overexpressed TOP2A, and in cells that overexpressed the TOP2A gene while exposed to NCTD. **(B)** The cell proliferation efficiency of different treatment HepG2 cells was examined using the CCK-8 assay. TOP2A overexpression attenuates NCTD-induced suppression of HCC cell proliferation **(C, D)** Flow cytometry was employed to quantify the apoptotic rate in different treatment HepG2 cells. TOP2A overexpression inhibits NCTD-induced apoptosis in HCC cells. **(C)** Flow diagram of apoptotic cells. **(D)** The result showed the apoptotic rate measured *via* flow cytometry in different groups **(E–H)** TOP2A overexpression reduces cell migration and invasion capabilities suppressed by NCTD in HepG2 cell, as demonstrated by wound healing **(E, F)** and transwell assays **(G, H)**. **(E)** The wound healing assay was performed to evaluate the migratory ability of HepG2 cells after NCTD exposure and TOP2A overexpression. Bars represent 100 µm. **(F)** Statistical plots compare cell migration efficiency in HepG2 cells. **(G)** Transwell invasion assay was performed to evaluate the migratory ability of HepG2 cells treated with NCTD and TOP2A overexpression. Bars represent 100 µm. **(H)** Statistical plots compare cell invasion efficiency. Bars represent 100 µm. **(I, J)** The cell cycle was analyzed by flow cytometry, TOP2A overexpression rescues NCTD-mediated cell cycle arrest in HCC cells. **(I)** Flow diagram of cell cycle, **(J)** Cell cycle statistical analysis diagram. Data are presented as mean ± SD. Statistical significance is indicated as *p < 0.05, **p < 0.01, ***p < 0.001, and ****p < 0.0001. All experiments were performed in triplicate.

We conducted further studies to determine whether the inhibitory effect of NCTD on the biological activities is mediated through targeting TOP2A in HCC. To achieve this, we overexpressed TOP2A in NCTD-treated HCC cells for rescue experiments (Figure 4A), and subsequently performed cell proliferation assays. The results revealed that the reduction in cell proliferation induced by NCTD was significantly reversed by TOP2A overexpression in HCC cells (Figure 4B). Additionally, TOP2A overexpression suppressed the NCTD-induced increase in cell apoptosis, as evidenced by the results of apoptosis assays (Figures 4C,D). Moreover, the results of wound healing and transwell assays demonstrated that the inhibitory effect of NCTD cell migration and invasion was reduced by TOP2A overexpression in HCC (Figures 4E–H). Furthermore, the cell cycle analysis indicated that TOP2A overexpression reversed the inhibitory effect of NCTD on the cell cycle arrest (Figures 4I,J). Collectively, these findings suggest that TOP2A overexpression could diminish the inhibitory effect of NCTD on the progression of HCC cells. TOP2A is a crucial therapeutic target for NCTD in HCC.

### NCTD-mediated downregulation of TOP2A is involved in cell cycle regulation

**FIGURE 5:**
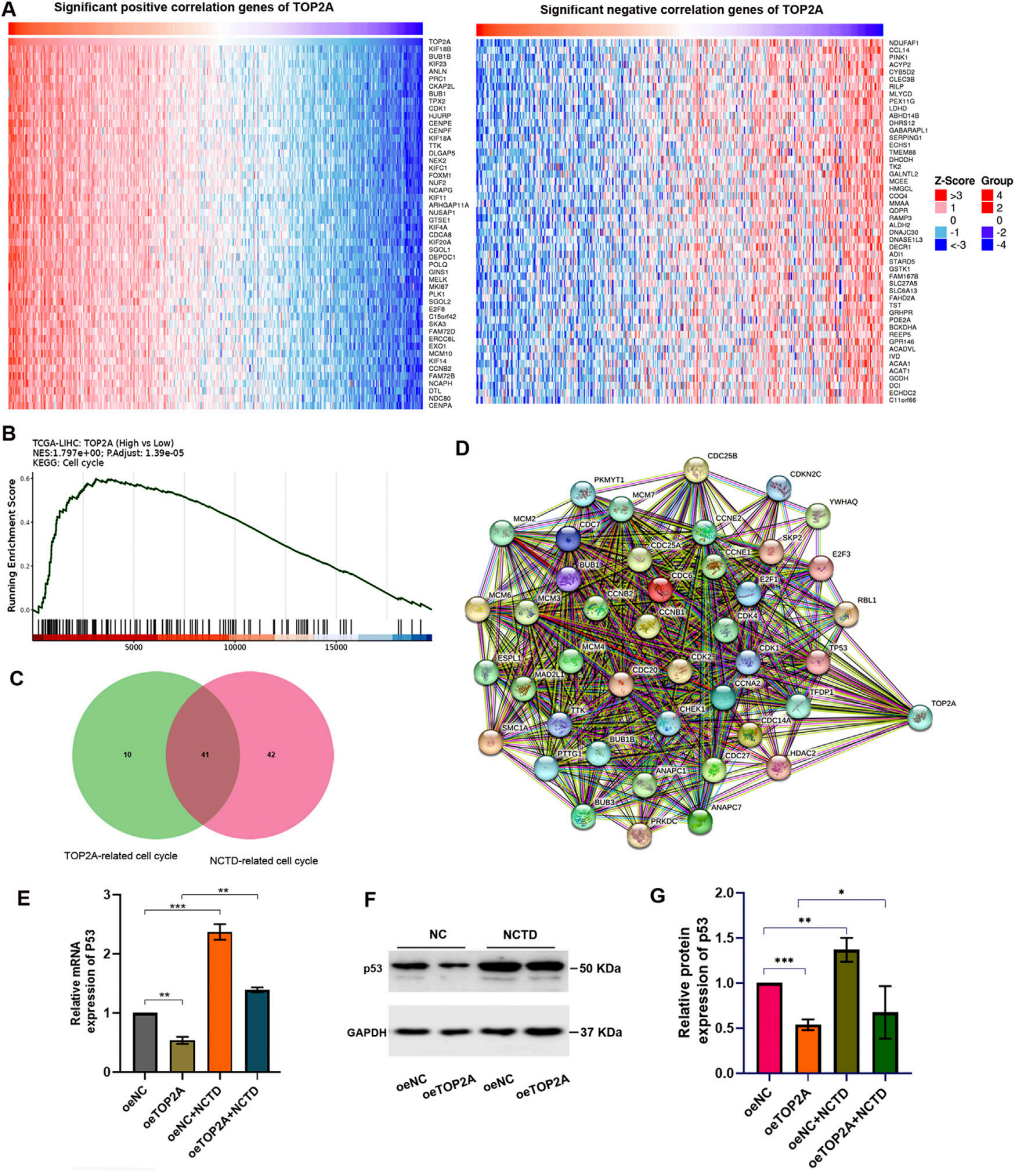
The NCTD-induced downregulation of TOP2A contributes to cell cycle regulation by modulating p53 expression. **(A)** Heatmap visualization of the correlation between TOP2A expression and its co-expressed genes in LIHC. **(B)** KEGG pathway enrichment analysis of TOP2A-associated genes, revealing significant biological pathways. **(C)** Venn diagram demonstrating the intersection of NCTD-responsive genes and TOP2A-associated genes, with particular emphasis on cell cycle regulatory components. **(D)** PPI network analysis of the overlapping genes identified in **(C)**, illustrating potential functional relationships. **(E)** Quantitative PCR analysis showing p53 mRNA expression profiles in HepG2 cells under three conditions: NCTD treatment, TOP2A overexpression, and combined NCTD treatment with TOP2A overexpression. **(F)** Western blot analysis of p53 protein expression in HepG2 cells under three experimental conditions: NCTD treatment, TOP2A overexpression, and combined treatment. **(G)** Densitometric quantification of p53 protein levels normalized to GAPDH loading control. All the Data were shown as mean ± SD. Statistical significance is indicated as *p < 0.05, **p < 0.01, and ***p < 0.001. All experiments were repeated three times.

To elucidate the molecular mechanisms by which NCTD modulates HCC phenotypes through TOP2A regulation, we conducted a comprehensive analysis of TOP2A-associated mRNA expression profiles in LIHC. Our analysis identified a spectrum of TOP2A-related co-expressed genes, including both positively and negatively correlated genes, as illustrated in Figure 5A. Subsequent KEGG pathway enrichment analysis demonstrated that these DEGs were significantly enriched in cell cycle-related pathways (Figure 5B), which exhibited remarkable consistency with the KEGG pathway analysis of CTD-related DEGs in HCC (Yan et al., 2023a). These findings strongly support our hypothesis that NCTD exerts its anti-HCC effects through TOP2A-mediated regulation of cell cycle signaling pathways.

Furthermore, our analysis identified cell cycle-related genes that showed significant associations with both NCTD treatment and TOP2A expression. We obtained a total of 41 intersection genes (Figure 5C). PPI network analysis revealed that TOP2A serves as a central hub node within the cell cycle-related gene network, demonstrating extensive interactions with other regulatory components. Of particular interest, our analysis identified the tumor suppressor gene p53 as a key network hub among TOP2A-related genes enriched in cell cycle pathways (Figure 5D). To validate the functional relationship between NCTD-mediated TOP2A regulation and p53 expression in HCC, we performed *in vitro* experiments using HepG2 cells. Our results demonstrated that NCTD treatment significantly upregulated p53 expression, while TOP2A overexpression attenuated this effect in NCTD-treated HCC cells (Figures 5E–G). These findings collectively suggest that NCTD-mediated downregulation of TOP2A enhances p53 expression, thereby contributing to cell cycle regulation during HCC treatment.

### NCTD regulates TOP2A expression through H3K27me3-mediated transcriptional repression

**FIGURE 6:**
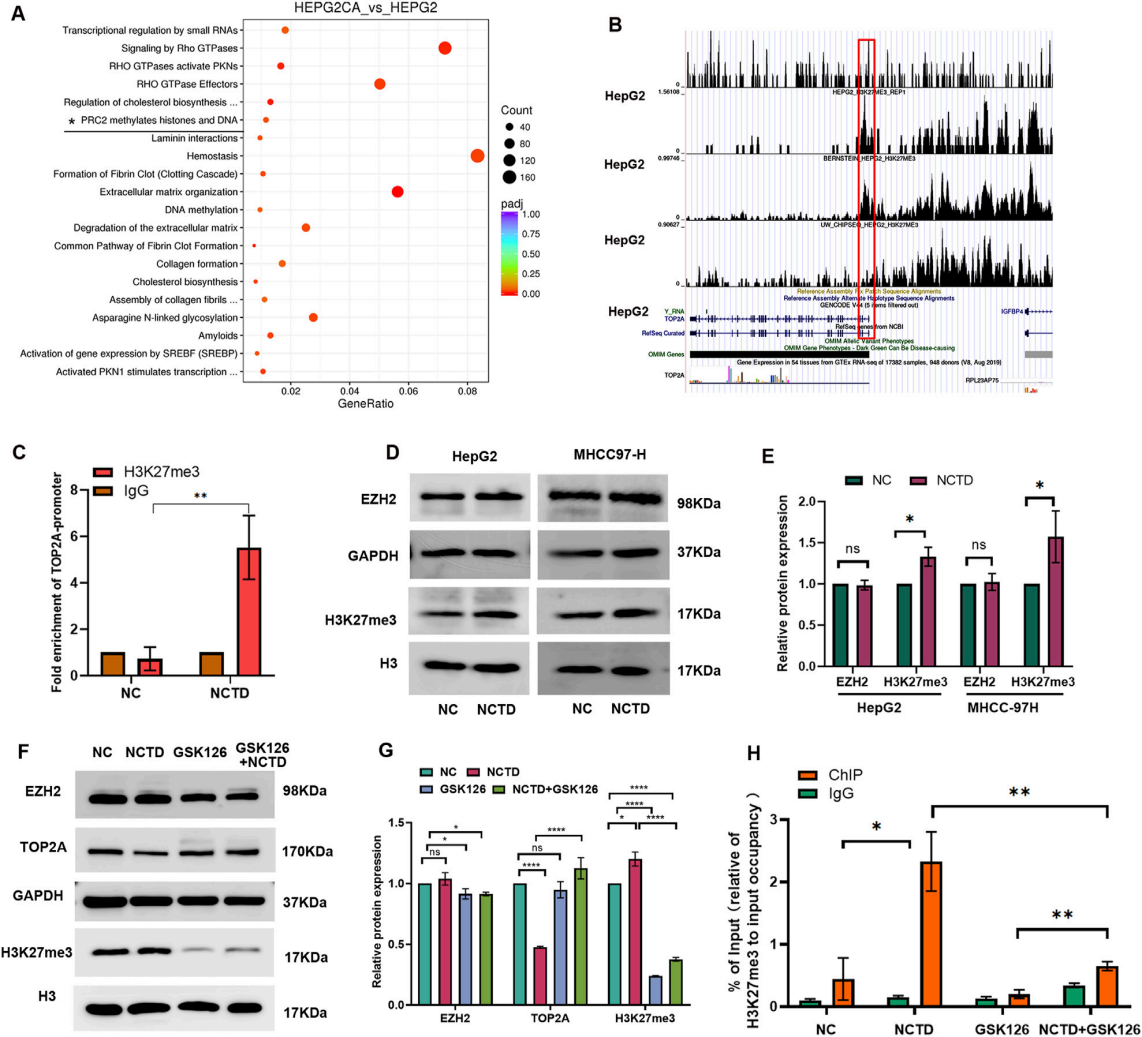
NCTD modulates TOP2A expression through H3K27me3-mediated transcriptional repression. **(A)** The bubble diagram shows that pathway enrichment analysis result. It demonstrates that CTD-related DEGs are significantly enriched in PRC2-mediated histone methylation pathways. **(B)** Genomic localization analysis reveals H3K27me3 enrichment at the TOP2A promoter region in HepG2 cells through ENCODE ChIP-seq data and UCSC Genome Browser visualization. **(C)** ChIP-qPCR validation of H3K27me3 occupancy at the TOP2A promoter in negative control (NC), and NCTD-treated HepG2 cells. IgG as the control. **(D)** Western blot analysis of protein expression of EZH2 and H3K27me3 in NCTD treated HepG2 cell. **(E)** Densitometric quantification of protein levels protein. EZH2 level was normalized to GAPDH, and H3K27me3 level was normalized to H3. **(F)** Rescue experiments demonstrating TOP2A expression recovery upon GSK126 treatment. Western blot analysis of protein expression of EZH2, TOP2A and H3K27me3 in different group, including NC group, NCTD-treated group, NCTD and GSK126 combination group. **(G)** Quantitative analysis of the band intensity of proteins was performed, EZH2 and TOP2A levels were normalized to GAPDH, and H3K27me3 level was normalized to H3. **(H)** ChIP-qPCR validation of H3K27me3 occupancy at the TOP2A promoter in NC, NCTD-treated HepG2 cells, NCTD and GSK126 were treated in combination. IgG as the control. All the Data were shown as mean ± SD. *p < 0.05,**p < 0.01 and ****p < 0.0001. All experiments were repeated three times.

In our previous study, we discovered that the differentially expressed genes associated with CTD were enriched in the H3K27me3 pathway (Yan et al., 2023a). Furthermore, we validated that the EZH2/H3K27me3-related cell cycle pathway serves as a therapeutic target for CTD in antitumor treatments (Yan et al., 2023b). In this study, our further pathway enrichment analysis using Reactome database also indicated that these genes were also associated with PCR2 methylates histones and DNA (Figure 6A). Subsequently, we analyzed the H3K27me3 occupying region in TOP2A gene based on H3K27me3 methylation-related chromatin immunoprecipitation (ChIP)-seq data from the Cistrome database for HepG2 liver cancer cell lines (Figure 6B). Additionally, we conducted ChIP-qPCR assays to confirm enrichment level of H3K27me3 in TOP2A at the NCTD-treated HCC cells. The results showed that the H3K27me3 level was increased in the TOP2A promoter region after NCTD treatment (Figure 6C). Based on these findings, we hypothesized that the NCTD-induced downregulation of TOP2A expression might be dependent on H3K27me3 related epigenetic transcription suppression.

EZH2 is enzymatic catalytic subunit of PRC2 that could alter downstream target genes expression. To confirm that whether the inhibition of TOP2A by NCTD is dependent on EZH2, the protein level of EZH2 was detected in NCTD treated HCC cell. Interesting, the protein level of EZH2 did not significantly increased, while H3K27me3 level was slightly increased after NCTD treatment (Figures 6D,E). To further unravel that NCTD regulated TOP2A expression was indeed attributed to H3K27me3, GSK126 was applied to reprogram the epigenetic pathways of H3K27me3 in HepG2 cell. As expected, GSK126 significantly inhibited the level of H3K27me3, and it effectively increased NCTD-induced TOP2A expression in HCC (Figures 6F,G). The occupancy of H3K27me3 in TOP2A was detected in GSK126 and NCTD treated HepG2 cells. The results showed that The NCTD-induced elevation of H3H27me3 in TOP2A was partially restored by GSK126 (Figure 6H). In addition, we transfected a siRNA targeting EZH2 into NCTD-treated HCC cells. In NCTD treated HepG2 cell, the expression of TOP2A was partial reversed by the downregulation of EZH2 in NCTD-treated HCC cells (Supplementary Figure 3A, B). These findings suggest that NCTD may suppress the expression of the TOP2A gene through H3K27me3-mediated transcriptional repression.

### NCTD targets EZH2 and affects the interaction between EZH2 and PP1

It is interesting that the expression of TOP2A was not significantly inhibited in GSK126 treated HepG2 cells, while GSK126 treatment restored the inhibitory effect of NCTD on TOP2A protein expression in HCC. Moreover, the clinical data analysis showed that EZH2 was positively correlated with TOP2A, which was significantly increased in liver cancer. Taken together, we speculated that the inhibitory effect of NCTD on TOP2A expression was not caused by the alteration of EZH2 expression in HCC.

**FIGURE 7:**
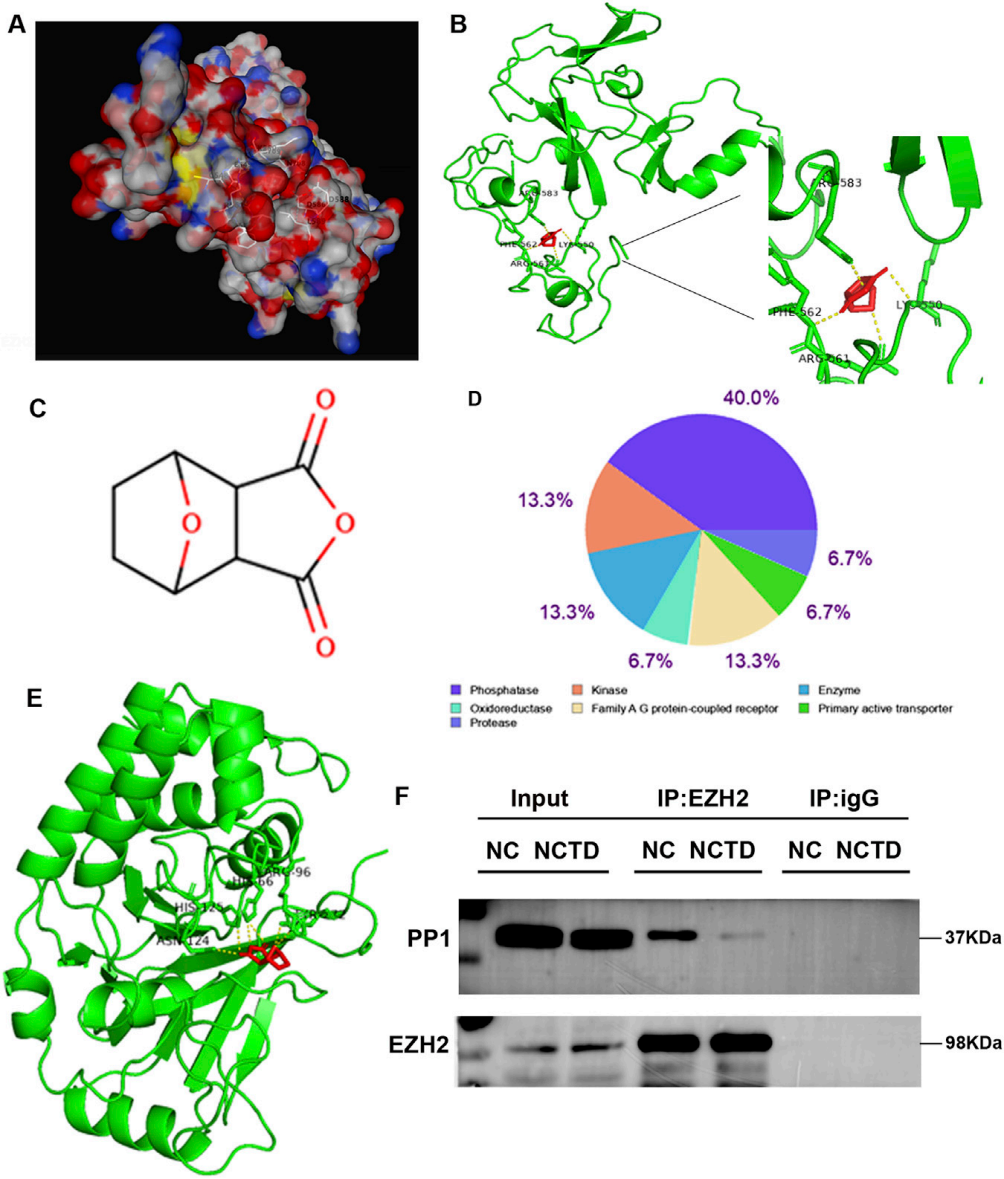
NCTD disrupts the EZH2-PP1 phosphatase interaction. **(A, B)** Molecular modeling and interaction analysis are conducted to investigate the binding of NCTD to EZH2. **(A)** Three-dimensional structural representation of the optimal docking conformation, with NCTD (white stick model) positioned in the EZH2 binding pocket. **(B)** Two-dimensional ligand-receptor interaction map depicting key binding residues (green) and NCTD (red) with hydrogen bonds (yellow dashed lines). **(C)** Chemical structure of NCTD. **(D)** Target class profiling of NCTD showing predominant interactions with phosphatases (40%), kinases (13.3%), and enzymes (13.3%). **(E)** Competitive binding analysis demonstrating NCTD’s preferential affinity for PP1 through comparative molecular docking studies. **(F)** Co-immunoprecipitation validation of EZH2-PP1 complex disruption both in the presence and absence of NCTD treatment. All experiments were independently replicated three times to ensure reproducibility.

Furthermore, we calculated the affinity values between the EZH2 protein and the small molecule NCTD. The results revealed that NCTD component exhibits strong affinity for EZH2, as confirmed by molecular docking analysis (Figures 7A,B), suggesting that EZH2 may be a pivotal target of NCTD in the treatment of HCC. The molecular docking results further indicated that NCTD likely bind to the amino acid region spanning positions 550 to 590 of EZH2. Therefore, we speculated that NCTD is not directly targeted to regulate EZH2 accumulation to suppress TOP2A expression in HCC.

In our current investigation, we employed computational approaches to predict potential molecular targets of NCTD (Figure 7C). Subsequent functional annotation revealed that approximately 40% of the predicted targets belong to phosphatase, 13.3% is kinase (Figure 7D). Furthermore, molecular docking analysis demonstrated that the particularly strong binding affinity was found between NCTD and protein phosphatase PP1 (Figure 7E). To further elucidate the molecular mechanism underlying NCTD-mediated regulation of EZH2, we conducted co-immunoprecipitation (Co-IP) assay to examine the physical interaction between PP1 and EZH2. The results showed that EZH2 could interact with PP1, and this interaction is weakened in HCC cells treated with NCTD (Figure 7F). Collectively, These findings collectively suggest that NCTD exerts its anti-tumor effects *via* directly targeting to EZH2 and modulating the interaction between EZH2 and PP1, thereby potentially influencing tumor progression pathways in HCC.

## Discussion

In recent year, the active ingredients derived from traditional Chinese medicine have garnered significant attention for their potential clinical applications in treating various cancers. Cantharidin and its derivatives NCTD have been confirmed to exhibit anti-tumour effect, particularly in liver cancer. NCTD has demonstrated multifaceted pharmacological actions with significant translational potential. Recent studies reveal that NCTD enhances sorafenib antitumor efficacy in HCC (Yousef et al., 2023). The lactoferrin functionalized polyethylene glycol liposomes loaded with norcantharidin can be used for targeted therapy of HCC (Mu et al., 2025). More innovatively, advanced delivery systems employing NCTD-loaded liposome-emulsion hybrids have shown remarkable potential to potentiate α-PD-1/L1 checkpoint immunotherapy (Liu et al., 2022). Given the promising application prospects of NCTD in liver cancer treatment, it is imperative to delve deeper into its therapeutic role. In this study, we investigated the mechanism underlying the transcriptional repression induced by NCTD in HCC. Specifically, we focused on the molecular mechanism by which it is involved in PRC2-mediated epigenetic regulation to inhibit tumor progression.

In this study, we revel that NCTD targets TOP2A to inhibit its expression, thereby inhibiting tumor progression in HCC. It have been reported that the elevated expression of TOP2A promotes the proliferation, metastasis, and invasion of HCC cells both *in vitro* and *in vivo*. It enhances the migration and invasion of HCC cells through the p-ERK1/2/p-SMAD2/snail pathway (Dong et al., 2021). In this study, we demonstrated that TOP2A is overexpressed in tumor tissues and associated with prognosis, including in HCC. These findings highlight TOP2A as a potential therapeutic target for HCC. Notably, we observed that NCTD inhibits the proliferation, metastasis, and invasion of HCC cells by downregulating TOP2A expression, suggesting that TOP2A may be a target of NCTD in HCC therapy. Therefore, a decrease in TOP2A expression in NCTD-treated HCC cells leads to the inhibition of HCC progression. In conclusion, our findings contribute to the understanding of the mechanisms by which NCTD exerts its antitumor effects in HCC, particularly through its targeting of TOP2A.

Many studies have confirmed that TOP2A plays a crucial role in mitotic chromosome condensation and segregation in cell cycle progression. It facilitates the progression of the cell cycle from the G2 to M phase by inhibiting CHK1 phosphorylation (López et al., 2025). Specifically, inhibition of TOP2A has been shown to suppress proliferative capacity and induce cell cycle arrest at G2 phase *via* p53 activation and CHK1 phosphorylation (Wang T. et al., 2022; Xiong et al., 2018). Our findings align with previous research demonstrating that the expression of TOP2A was associated with the p53 to modulate cell cycle pathway. Furthermore, we found that the NCTD-mediated inhibition of TOP2A expression leads to significant suppression of HCC progression *via* regulating p53 associated cell cycle pathway. Therefore, by targeting TOP2A, NCTD appears to effectively disrupt the cell cycle progression of cancer cells while simultaneously activating tumor suppressor pathways. The observed effects of NCTD in suppressing HCC progression further support this hypothesis.

An increasing number of studies suggest that TCM holds potential as a reliable source of epigenetic drugs, capable of balancing various epigenetic modifications (Wang et al., 2024). Curcumin, a component of TCM, has been shown to decrease the expression of the PRC2 subunit EZH2 in numerous cancer cells. Similarly, berberine has been found to upregulate the expression of histone demethylase KDM6A and histone methyltransferase SETD7, while downregulating histone methyltransferases WHSC1I, WHSC1II, and SMYD3 to reduce the expression levels of H3K4me3, H3K27me3, and H3K36me3 (Khan et al., 2024; Wang et al., 2016). Despite these promising findings, the influence of TCM on histone methylation remains somewhat limited.

Our study elucidates a novel epigenetic mechanism by which NCTD suppresses HCC progression. We focus on a significant correlation between NCTD-related TOP2A regulation and PRC2, a chromatin-associated methyltransferase that catalyzes the methylation of lysine 27 on histone H3. We hypothesize that epigenetic regulation plays a pivotal role in modulating TOP2A expression, a notion supported by numerous studies. For instance, recent research has revealed that USP7 stabilizes KDM5B and promotes cisplatin resistance through the ZBTB16/TOP2A axis (Zhang et al., 2024). Additionally, TOP2A is upregulated by WHSC1-mediated H3K36me2, which activates the PI3K/AKT signaling pathway in HCC (Zhong-Ming et al., 2022). Moreover, EZH2 enhances TOP2A expression by epigenetically silencing miR-139–5p through H3K27me3, thereby influencing cellular senescence and proliferation in HCC (Wang et al., 2023). Consistent with these results, we observed a significant increase in H3K27me3 levels within the TOP2A promoter region following NCTD treatment, suggesting that the suppression of TOP2A expression by NCTD is mediated by the enrichment of repressive chromatin marked by H3K27me3 in HCC.

EZH2, the catalytic subunit of PRC2, plays a crucial role in mediating histone trimethylation and inhibiting the expression of its target genes throughout the development and progression of HCC. While EZH2 is frequently overexpressed in HCC and correlates with poor prognosis. Moreover, EZH2 and TOP2A are highly co-expressed in HCC tumor tissues, clinically (Wang et al., 2023). We observed preservation of EZH2 protein levels despite significant TOP2A downregulation in HCC. Our findings suggest that NCTD modulates PRC2 activity through post-translational mechanisms rather than altering EZH2 expression. A series of reports also showed that the post-translational modification of EZH2 protein is essential for PRC2-associated methyltransferase activity. Phosphorylation is probably the most well-known process which modulates the molecular property and function of EZH2 (Minnebo et al., 2013; Zhang et al., 2022). In this study, we also found that the majority of NCTD targets were protein phosphatases and kinases. Moreover, particularly noteworthy is our discovery that NCTD could targets EZH2 and PP1 phosphatase, leads to disrupt the EZH2-PP1 interaction, potentially altering the phosphorylation landscape of PRC2 components. This may be the reason for TOP2A decrease after NCTD treatment in HCC. Additionaly, in the absence of NCTD treatment, GSK126 treatment did not significantly upregulated TOP2A expression, suggesting that there are no H3K27me3 binding peaks in TOP2A gene in HCC. This is consistent with previously result that the TOP2A transcription is not directly regulated by EZH2 in HCC (Wang et al., 2023).

**FIGURE 8:**
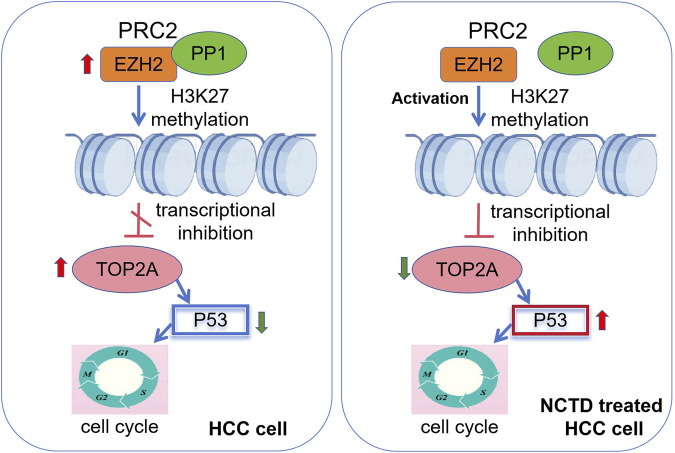
Epigenetic mechanism of NCTD in suppressing HCC progression. A schematic diagram depicting the regulatory mechanism. In HCC, overexpression of EZH2 and TOP2A drives oncogenic progression. EZH2 and TOP2A are highly expressed in HCC. EZH2 interacts with PP1, limiting its PRC2 methyltransferase activity. On the other hand, the elevated TOP2A expression suppresses p53 to regulate cell cycle, and ultimately facilitate the progression of HCC. In NCTD treatment, NCTD targets PP1 and EZH2, thereby disrupting their physical interaction. This disruption results in the liberation of PRC2, enhancing its methyltransferase activity of EZH2. Consequently, there is epigenetic silencing of TOP2A through the mediation of H3K27me3. The ensuing epigenetic silencing of TOP2A activates the p53 pathway and contribute significantly cell cycle arrest, leading to the suppression of HCC progression.

In conclusion, our study unveils a novel epigenetic pathway through which NCTD exerts its anti-tumor effects in HCC. Based on these findings, we propose a mechanistic model the physical interaction between EZH2 and PP1 creates a regulatory constraint, limiting EZH2’s accessibility to histone substrates and consequently reducing H3K27me3 deposition at TOP2A. Thus, the expression of TOP2A was abnormally increased in HCC. This molecular constraint provides a mechanistic explanation for the concurrent overexpression of both EZH2 and TOP2A observed in HCC tissues, despite EZH2’s well-characterized role as a transcriptional repressor. While, in NCTD treatment HCC cell, NCTD simultaneously targets PP1 and EZH2, thereby releasing EZH2 from PP1-mediated inhibition. This liberation enhances PRC2’s methyltransferase activity, leading to increased H3K27me3 deposition specifically at the TOP2A promoter region. The resultant epigenetic silencing of TOP2A likely contributes to cell cycle arrest and suppression of HCC progression (Figure 8). However, whether NCTD-induced H3K27me3 enrichment is TOP2A specific or affects a wider range of PRC2 target genes remains to be explored. Moreover, it is crucial to emphasize that the precise mechanism by which NCTD modulates EZH2 phosphorylation status remains unexplored. Importantly, future research will be crucial to comprehensively elucidate the molecular mechanisms that underlie the epigenetic effects of NCTD. Taken together, these converging evidence from molecular pathway modulation will provide a robust scientific rationale for prioritizing NCTD in HCC treatment.

## Materials and methods

### Cell culture and reagents

NCTD was procured from Aladdin Chemical Company, located in China, with a CAS number of 5,442–12-6 and a guaranteed purity of ≥98%. The HCC cell lines, HepG2 Huh7 and MHCC97-H, were sourced from the Cell Bank of the Chinese Academy of Sciences and were authenticated through short tandem repeat (STR) profiling to ensure their identity. All experimental procedures were conducted using cells within the early passages (<10) of each respective cell line to maintain genetic stability. The cells were maintained in Dulbecco’s Modified Eagle Medium (DMEM) (Gibco, United States), supplemented with 10% fetal bovine serum (FBS) (Gibco, United States), 100 μg/mL streptomycin, and 100 U/mL penicillin (Sigma, United States). The cultures were incubated at 37 °C in a humidified atmosphere containing 5% CO_2_.

### Plasmid construction and lentiviral infection

TOP2A overexpression vector was purchased from Yunzhou Biological Company (Vector builder, China). The human TOP2A gene was cloned into the plv [Exp]-EGFP/Puro-EF1A vector to facilitate transient transfection. For the production and infection of viruses, the plasmids were transfected into HEK 293T cells for a duration of 48 h. Subsequently, the culture medium was harvested and enriched with polybrene at a concentration of 10 μg/mL. This enriched medium was then incubated with HCC cells for an additional 48 h, followed by selection with puromycin at a concentration of 2 μg/mL to establish stable cell lines.

The si-EZH2 and si-NC were synthesized by Vector builder, Guang zhou, China. To achieve knockdown of endogenous EZH2, siRNA duplexes specifically targeting EZH2, along with a negative control siRNA (both at a concentration of 100 nM), were transfected into HCC cells using RNAiMax reagent (Invitrogen; Thermo Fisher Scientific, Inc., Waltham, MA, United States) for a period of 48 h.

### Cell proliferation assay

Cell proliferation was assessed using the MTS assay. Specifically, cells were seeded at a density of 1 × 10^3^ cells per well in a 96-well plate. Following incubation periods of 24 h, 48 h, 72 h, and 96 h, the cells were exposed to medium containing 20 μL of MTS reagent at a concentration of 0.5 mg/mL for 3 hours. The absorbance was then measured at a wavelength of 490 nm using the EnSpire Multimode Plate Reader (PerkinElmer).

### Cell apoptosis assay

The cells were collected and processed using the FITC Annexin V apoptosis detection kit I (BD Biosciences Pharmingen, United States), following the manufacturer’s guidelines meticulously. Immediately thereafter, the samples were subjected to flow cytometry analysis utilizing the BD Canto II system (United States). The gathered data were then analyzed with FlowJo 10 software (BD Canto II, United States).

### Cell cycle assay

A total of 5 × 10^6^ cells were fixed in 500 μL of 70% cold ethanol at 4°C for a period of 2 h. Following fixation, the cell pellet was thoroughly washed with phosphate-buffered saline (PBS) and then incubated with 100 μL of RNaseA in a 37°C water bath for 30 min. Subsequently, the cells were stained using a propidium iodide (PI)/RNase staining buffer (sourced from BD Biosciences Pharmingen, United States), adhering strictly to the manufacturer’s instructions. The stained cells were analyzed using a flow cytometer, with the red fluorescence intensity recorded at a wavelength of 488 nm. The acquired data were meticulously analyzed using FlowJo 10 software (BD Canto II, United States).

### Wound healing assay

A total of 5 × 10^6^ cells were seeded into 6-well plates. Once the cells had reached a confluence level of 90%, they were scratched using 100 μL pipette tips to create three parallel lines. Subsequently, the cells were washed twice with PBS buffer. After allowing a wound to form for 24 h, the size of the wound was carefully measured and photographed. The images were then analyzed using ImageJ software. The wound healing rate was computed using the following formula: wound healing rate = [(scratch width at 0 h) - (scratch width at 24 h)]/(scratch width at 0 h) × 100 %.

### Transwell migration and invasion assay

The cells were plated in the upper chamber of transwell plates sourced from Corning (United States). The lower chamber of the transwell was supplemented with serum-free medium containing 10% FBS as a chemoattractant. Following an incubation period of 24 h for the migration assay and 48 h for the invasion assay, the cells that had successfully migrated or invaded through the membrane were stained with crystal violet for visualization and then counted.

### Real-time-PCR

Total RNA was extracted utilizing RNA Plus reagent sourced from Takara (China), followed by reverse transcription using the PrimeScript RT Reagent Kit with gDNA Eraser, also provided by Takara (China). The expression levels of the mRNA for each gene were subsequently confirmed through the use of the SYBR Premix Ex Taq II kit (Takara, China) on the Thermal Cycler CFX6 System manufactured by Bio Rad (United States). The primer sequences showed in Supplementary Table S1.

### Western blotting

Total proteins were extracted from cells using RIPA buffer containing protease inhibitors, and the protein concentrations were determined using a BCA protein assay kit (Thermo Fisher Scientific, Inc.). Each protein sample was resolved by 10% SDS-PAGE and transferred onto PVDF membranes. Following a blocking step with 5% nonfat milk for 1 h at room temperature, the membranes were incubated overnight at 4°C with primary antibodies specific to the target proteins of interest. After washing the membranes three times with TBST solution, they were incubated with horseradish peroxidase (HRP)-conjugated secondary antibodies for 1 h at room temperature. Protein bands were visualized using enhanced chemiluminescence (ECL) reagents from Millipore (United States) and imaged with an eBLOT Western Blot system (eBLOT, China).

For semi-quantitative analysis, densitometric measurements of the protein bands were performed using ImageJ software. The intensity of each band was normalized to that of GAPDH, a control protein, to account for variations in protein loading. The relative expression levels of the target proteins were then calculated and presented as fold changes compared to control samples. Each independent experiment was conducted in triplicate or more to ensure reproducibility and statistical significance. The information of antibody showed in Supplementary Table S2.

### Differential gene expression analysis based on RNA-seq data

The RNA-seq data in CTD treated HepG2 cell was obtained based on our previous study (Yan et al., 2023a), The“edger”R package was used to screen differentially expressed genes in our previous CTD associated RNA-seq data, and“limma”R package was used to screen DEGs. The threshold was set as log2FC < -2, and FDR <0.05 was considered significant. DEGs that met this criteria were chosen for further analysis. The construction of a hierarchical clustering analysis were also performed by the R packages“pheatmap”.

### Identification and functional enrichment analysis of hub genes related to CTD

The Venn diagram was employed to identify overlapping genes among the significant modules. The overlapping genes were selected as potential target genes of CTD. To further investigate the biological functions of hub genes, enrichment analyses of biological functions and pathways were conducted, which can be described and visualized using KEGG pathway enrichment analysis and gene set enrichment analysis.

### Hub gene expression and survival analysis

The differences in the expression of the hub genes related to CTD between tumor tissue and normal tissue were determined based on data from TCGA RNA-seq and available in UCSC Xena. Subsequently, the GSCA database was used to perform survival predication, including overall survival, progression free survival, disease specific survival, and disease free survival.

### Immunohistochemical (IHC) staining

The Human Protein Atlas database (Human Protein Atlas https://proteinatlas.org) was used to check the protein expression level of TOP2A (Minnebo et al., 2013). Immunofluorescence staining images were also used to show the expression and subcellular localization of TOP2A in HCC.

### Analysis of TOP2A co-expressed gene expression

In order to clarify the function of TOP2A, we analyzed the genes related to its expression, we selected the data set LIHC cohort, data type RNAseq, and the statistical method “Pearson correlation test”to analyze the co-expression genes of TOP2A in LIHC. The positively and negatively correlated genes were shown in a heat map.

### Chromatin immunoprecipitation (ChIP) -qPCR assay

ChIP assay was performed using a Simple ChIP Enzymatic Chromatin IP Kit (Magnetic Beads) (Cell Signaling Technology, #9003) following the manufacturer’s protocol. In brief, a total of 4 × 10^7^ cells were collected and fixed with formaldehyde (1% final concentration directly added to the culture media) for 10 min. This was followed by treatment with lysis extraction buffer containing a protease inhibitor cocktail. The cell lysates were then digested with MNase at 37 °C for 30 min to obtain chromatin fragments. Subsequently, ultrasound processing was performed to generate DNA fragments ranging from 100 to 500 bp. Primer sequences of ChIP-qPCR, F:TTCCCCTCGCTAACAACGTC, R: GCC​AAT​GAG​AAG​GGC​TCA​CT.

### Coimmunoprecipitation (Co‐IP)

The cells were lysed using IP buffer while kept on ice for a duration of 30 min, followed by the collection of proteins through centrifugation. Subsequently, the supernatants were incubated overnight at 4°C with either anti-EZH2 antibody (Santa Cruz) or IgG (Protein tech), along with Protein A/G beads (Santa Cruz, sc-2003). The following day, the beads bound to the target protein were thoroughly washed with IP buffer at least three times. After washing, the protein was evaluated using 2× SDS sample buffer for subsequent Western blot analysis.

### Protein-protein interaction (PPI) network construction

The PPI network of all identified protein was generated based on the STRING database. All interactions with a confidence level ≥0.7 (high confidence) were acquired. The Heatmap of interaction network was plotted by an online platform for data analysis and visualization (https://www.bioinformatics.com.cn).

### Docking verification

To investigate the potential binding interaction between NCTD and EZH2 or PP1, we conducted a docking analysis employing advanced molecular docking techniques. The three-dimensional (3D) structural data for NCTD were retrieved from the PubChem database, whereas the protein structure files for EZH2 were sourced from the Protein Data Bank (PDB) (http://www.rcsb.org/). Prior to docking, preprocessing of the structures was carried out using PyMOL (https://pymol.org/) software. Subsequently, semiflexible molecular docking simulations were performed using Auto Dock Vina (http://vina.scripps.edu/). The binding energy was utilized as a quantitative metric to assess the degree of docking, with a threshold of ≤ -5 kcal/mol being considered indicative of a feasible binding interaction.

### Statistical analysis

All statistical analyses were performed using GraphPad Prism 8 (GraphPad Software v8.0.2.236 San Diego, CA). Differences between the two groups were compared using a two-tailed unpaired Student’s t-test. Data were presented as Mean ± SD as indicated in the figure legends and the significance of differences was classified as *p < 0.05, **p < 0.01, ***p < 0.001, or ns (not significant). *p* values of <0.05 were considered statistically significant. All experiments were performed at least in triplicate.

## Data Availability

The original contributions presented in the study are included in the article/Supplementary Material, further inquiries can be directed to the corresponding authors.

## Supplementary material

SUPPLEMENTARY FIGURE S1 The cytotoxicity of NCTD in normal liver cells and different HCC cells. **(a-c)** The proliferation of HCC cell lines and normal liver cells treated with NCTD, which was assessed using the CCK-8 assay. NC stands for negative control, which was treated with 0.1% DMSO, L group: low concentrations of NCTD (20 µM). **(a)** In normal liver cell L02, **(b)** MHCC97-H cell, **(c)** Huh7 cell. All data are reported as mean ± SD. Significance levels are denoted as ****p* < 0.001. All experiments were conducted in triplicate to ensure reproducibility.

SUPPLEMENTARY FIGURE S2 The expression of TOP2A in normal liver cells and different HCC cells. **(a)** The mRNA expression of TOP2A in normal liver cells and different HCC cells. **(b)** The protein expression of TOP2A in normal liver cells and different HCC cells. **(c)** Quantitative analysis of protein band intensity of TOP2A normalized to GAPDH. All data are reported as mean ± SD. Significance levels are denoted as ***p* < 0.01, ****p* < 0.001, and *****p* < 0.0001. All experiments were conducted in triplicate to ensure reproducibility.

SUPPLEMENTARY FIGURE S3 Rescue experiments demonstrating TOP2A expression partial recovery following EZH2 knockdown**.** **(a)** Western blot analysis of protein expression of EZH2, TOP2A and H3K27me3 in different group, including NC group, NCTD-treated group, and siEZH2 transfection combined with NCTD treatment group. **(b)** Quantitative analysis of the band intensity of proteins was performed, EZH2 and TOP2A levels were normalized to GAPDH, and H3K27me3 level was normalized to H3. All data are reported as mean ± SD. Significance levels are denoted as **p* < 0.05, ***p* < 0.01, and ****p* < 0.001. All experiments were conducted in triplicate to ensure reproducibility.
