# Supplementary material for: Norcantharidin inhibits TOP2A expression via H3K27me3 mediated epigenetic regulation to alleviate the progression of hepatocellular carcinoma
Source: Front Pharmacol. 2025 Apr 3;16:1541298. doi: 10.3389/fphar.2025.1541298 (PMC12015943; doi:10.3389/fphar.2025.1541298)
Supplement: Supplementary file 1 [file Table1.docx]

**Table S1 Primer sequences**

| gene | primer | primer sequences |
| --- | --- | --- |
| TOP2A  EZH2  TP53 | Forward Primer  Reverse Primer  Forward Primer  Reverse Primer  Forward Primer  Reverse Primer | GAAGTGTCACCATTGCAGCC  CATGTCTGCCACCCTTGGAT  CAAAGCACAGTGCAACACCA  AGCGGCTCCACAAGTAAGAC  CCCTCCTCAGCATCTTATCCG  GCACAAACACGCACCTCAAA |

**Supplementary Table S1. Gene expression detection quantitative PCR primer sequence.**

**Table S2 Main antibody information**

| **Antibody** | **Experiment** | **source** |
| --- | --- | --- |
| GAPDH | WB | Proteintech (China) |
| H3 | WB | Abcam（USA） |
| H3K27me3 | WB/ ChIP | Abcam（USA） |
| TOP2A | Co-IP | Abcam（USA） |
| TOP2A | WB | Proteintech (China) |
| EZH2 | WB | Proteintech (China) |
| EZH2 | Co-IP | Abcam（USA） |
| PP1 | WB/ Co-IP | Abcam（USA） |

**Supplementary Table S2. Antibody information of experiment.**
