# Supplementary figures and images for: Norcantharidin inhibits TOP2A expression via H3K27me3 mediated epigenetic regulation to alleviate the progression of hepatocellular carcinoma
Source: Front Pharmacol. 2025 Apr 3;16:1541298. doi: 10.3389/fphar.2025.1541298 (PMC12015943; doi:10.3389/fphar.2025.1541298)

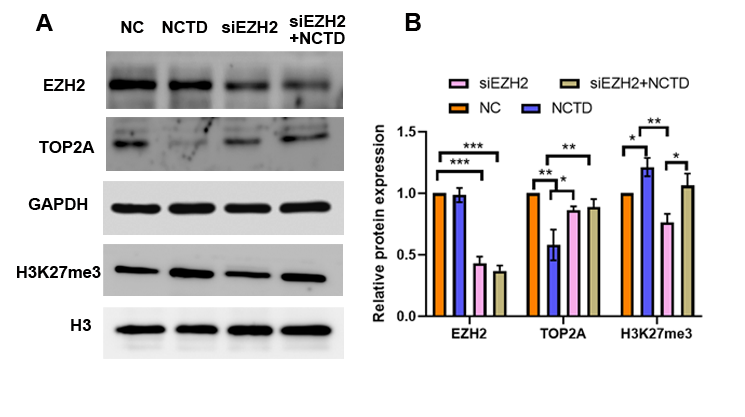

Supplement: Supplementary file 2 [file Image3.tif]

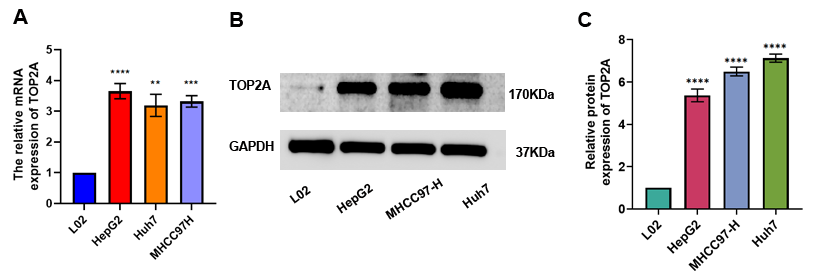

Supplement: Supplementary file 3 [file Image2.tif]

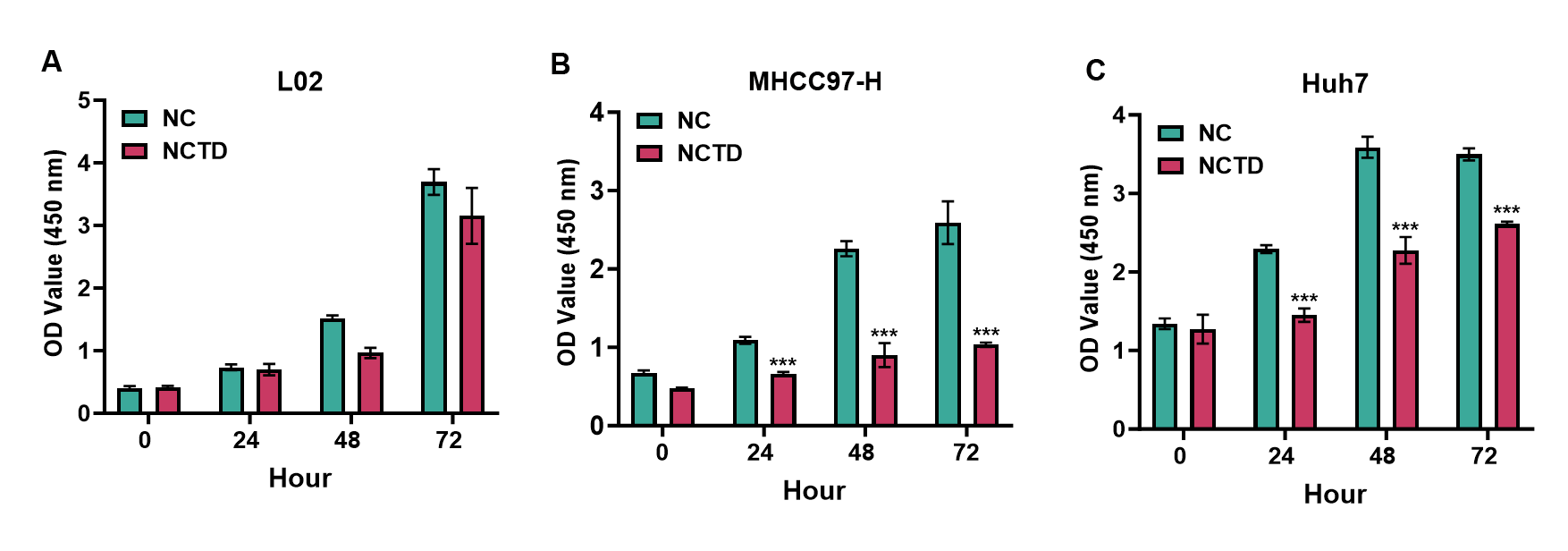

Supplement: Supplementary file 4 [file Image1.tif]
